# The use of transvaginal ultrasound alters physiologic uterine peristalsis in gynecologic participants

**DOI:** 10.1016/j.xfre.2024.06.004

**Published:** 2024-06-25

**Authors:** Kelsey Anderson, Sicheng Wang, Stephanie Pizzella, Qing Wang, Yong Wang, Valerie Ratts

**Affiliations:** a Department of Obstetrics and Gynecology, Washington University School of Medicine in St. Louis, St. Louis, Missouri; b Department of Electrical and Systems Engineering, Washington University in St. Louis, St. Louis, Missouri; c Mallinckrodt Institute of Radiology, Washington University School of Medicine in St. Louis, St. Louis, Missouri

**Keywords:** Peristalsis, transvaginal ultrasound, electrophysiologic imaging system

## Abstract

**Objective:**

To study whether transvaginal ultrasound (TVUS) affected the uterine peristalsis (UP) patterns in nonpregnant participants.

**Design:**

Institutional review board–approved, prospective observational cohort study. The noninvasive UP imaging (UPI) system uses electrode patches placed on the patient’s skin just above the pubic bone and on the low back to quantify the 3-dimensional electrical activation pattern during UP by calculating peristalsis frequency, duration, magnitude, and activation ratio. A 20-minute UPI scan was completed without TVUS followed by a 10-minute UPI scan acquired simultaneously during TVUS examination as a comparison.

**Setting:**

University medical center.

**Patient(s):**

Twenty-eight participants with regular menstrual cycles not taking hormonal medication and with a normal uterus were included in analysis.

**Intervention(s):**

Subjects were imaged longitudinally during the four phases of the menstrual cycle (menses, proliferative, periovulatory, and secretory) with a UPI scan followed by concurrent TVUS and UPI scan. Serum hormone levels (estradiol and progesterone) and TVUS evaluating follicular development were obtained during each visit to confirm menstrual cycle phase.

**Main Outcome Measure(s):**

Duration, frequency, magnitude, and activation ratio of the UP waves.

**Result(s):**

With the use of simultaneous TVUS, UP waves had a change in at least one of the outcomes measured in all visits. The frequency, magnitude, and duration were significantly higher with TVUS use in all phases of the menstrual cycle. The activation ratio was higher with TVUS during all phases except the periovulatory phase.

**Conclusion(s):**

This study demonstrated that TVUS may inherently affect UP waves. Therefore, noninvasive technology may more accurately measure physiologic peristalsis waves.

Studies have documented spontaneous, mild peristalsis waves or contractions from the inner layer of the myometrium (stratum subvasculare), which is distinct from labor contractions occurring at all layers of the myometrium (1, 2, 3, 4, 5, 6). Although the uterus is quiescent in the prepubertal and menopausal stages, peristalsis waves are vital as well as dynamic during the reproductive lifespan, specifically during the four phases of the menstrual cycle. During menstruation, peristalsis waves predominantly move from the fundus to the cervix to expel blood/endometrial tissue. A transition then occurs during the proliferative phase where cervix-to-fundus contractions dominate to facilitate active sperm transport during the periovulatory phase. Lastly, during the secretory phase, a more bidirectional peristalsis pattern occurs to not expel the embryo and to facilitate implantation (5, 7). Imaging studies using ultrasound (US) and intrauterine pressure catheters have provided evidence that the pattern, direction, as well as frequency of these contractions vary throughout the phases of the menstrual cycle (1, 4, 8, 9). To study the uterine peristalsis (UP) noninvasively, we developed a novel high-resolution electrophysiologic imaging system called UP imaging (UPI) to image the UP waves objectively and quantitatively (7).

Ultrasound technology uses high-frequency sound pulses to create images and has been a staple in obstetrics and gynecology for many decades (3, 5, 10, 11). Sonograms are part of most facets of gynecologic care including to monitor pregnancies, diagnose pathology, and, with assisted reproductive technology, perform procedures such as oocyte retrievals as well as embryo transfers. However, biologic effects, especially at a tissue/organ level, have not been well studied (2).

Ultrasound uses energy in the form of sound waves that are transmitted into the body, and waves echoed back are recorded to produce an image. Both thermal and nonthermal effects could explain the changes observed with transvaginal ultrasound (TVUS) use. Sound waves can be converted into heat, and this process has clinical application for procedures such as ablations; however, it can also occur unintentionally with transducers. The use of a transvaginal probe also causes direct pressure on the cervix and indirect pressure on the uterus creating mechanical (nonthermal) effects (11). This is more likely to explain peristalsis changes given that postpartum uterine massage is well documented to physically cause contractions from the release of prostaglandins (12, 13). To a lesser extent, a similar mechanical mechanism may be occurring in gynecologic participants undergoing TVUS through direct pressure of the probe on the cervix and indirectly on the uterus.

To study this possible effect, we employed the thoroughly developed and validated UPI system (7, 14, 15, 16, 17) for the longitudinal assessment of UP waves. Previous validation of the UPI system found that it measures peristalsis waves to the same accuracy as US but with advantages including being noninvasive and objective (7). Our system measures myometrial electrical activity quantitatively and objectively in a noninvasive manner. The UPI system combines a magnetic resonance imaging (MRI) scan to determine body-uterus geometry and body surface electrodes to determine body surface potential placed into a software system to provide reconstructed uterine surface potentials. Magnetic resonance imaging alone has been used to study peristalsis waves because it is noninvasive; however, it cannot detect the magnitude of waves and is not scalable. Here, we examined the effect of TVUS on UP waves using the noninvasive UPI system.

## Materials and methods

### Ethics statement

All UPI scans with TVUS examinations were performed at the Center for Outpatient Health and Division of Reproductive Endocrinology and Infertility, Department of Obstetrics and Gynecology, at the Washington University in St. Louis with approval by the Institutional Review Board.

### Patient enrollment and human study

Nonpregnant participants between the ages of 18 and 37 years were recruited for this study. Participants were defined as subjects with regular, predictable menstrual cycles every 24–35 days. Participants who were postmenopausal, pregnant, or breastfeeding, had a uterine anomaly, or had an exposure to medication known to affect uterine contractility (i.e., magnesium, opioids, beta antagonists, and nifedipine) were excluded from this study. In addition, participants whose abdominal circumference was >55 cm or had MRI contraindications (e.g., pacemaker and metal implants) were excluded from this study as well. Twenty-eight eligible participants were enrolled into this study after signing an informed consent. Each patient was imaged longitudinally at four phases of the menstrual cycle (menses, proliferative, periovulatory, and secretory). Supplemental Figure 1 (available online) demonstrates patient recruitment and enrollment flow in this study.

### Determination of phase in the menstrual cycle

The phase of menstrual cycle was determined by agreement with two clinicians who considered the following: cycle day (CD) 1 of their menstrual period before study visit and after study completion; CD; cycle length; estradiol (E2) and progesterone (P4) levels; US findings; and report of onset of positive luteinizing hormone (LH) surge. Menses was assigned during the presence of menstrual bleeding until day 5. The early follicular/midfollicular phase was assigned when with the following conditions: CD6–CD11; an E2 level of <160 pg/mL; a P4 level of <3.0 ng/mL; no LH surge reported; and/or lead follicle measuring <16 mm. The late follicular/periovulatory phase was assigned with the following conditions: within 14–16 days from the final reported menstrual period (CD1) at the end of the study period; an E2 level of >160 pg/mL; a P4 level of <3.5 ng/mL; lead follicle measuring >16 mm on US; and/or within 0–2 days of reported LH surge. The luteal phase was assigned when with the following conditions: within 12–13 days from the onset of the next reported menstrual period (CD1); >3 days from reported LH surge; a P4 level of >3.5 ng/mL; and/or US revealing new-onset complex ovarian cyst consistent with a corpus luteum.

### MRI scan

**Figure 1:**
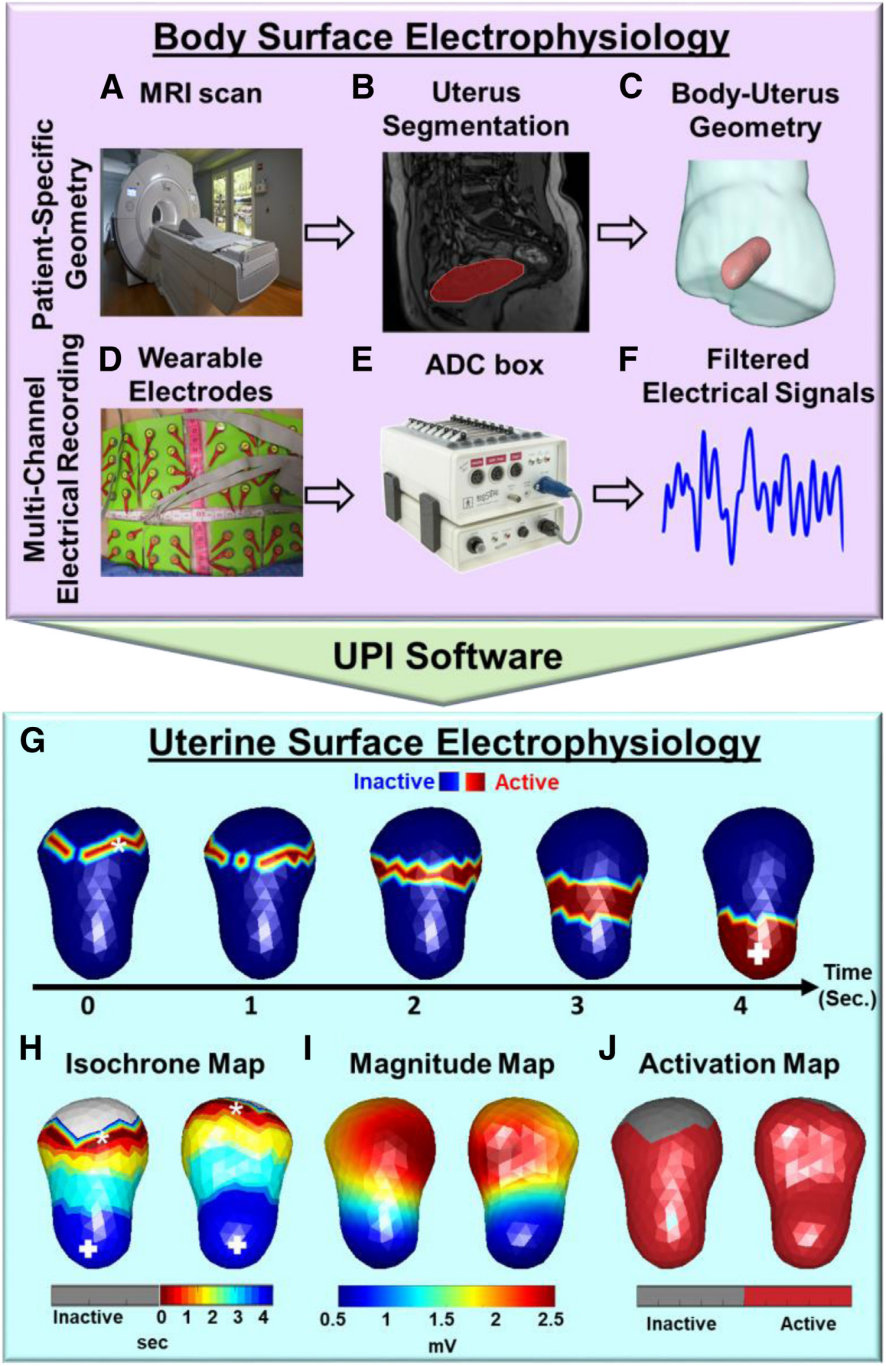
Ultrasound imaging system with automatic peristalsis detection and multiparametric quantification. (**A**) T1-weighted anatomical magnetic resonance imaging (MRI) scan. (**B**) Magnetic resonance imaging segmentation. (**C**) Magnetic resonance imaging–constructed body-uterus geometry. (**D**) Wearable electrode patches. (**E**) Multichannel ADC box. (**F**) Filtered slow-wave electrical signals (bandwidth, 0.01–0.05 Hz). (**G**) Electrical activation sequence of one peristalsis wave from the fundus to the cervix, with the red color representing the regions experiencing waves. (**H**) Isochrone map, with the gray, red, and blue colors representing inactive, early, and late activation regions, respectively. (**I**) Magnitude map showing the distribution of electrical potential. (**J**) Activation map showing the electrically activated uterine region in red. Figure 1 is adapted from the existing work (15–17), with modifications. ADC = analogue-to-digital converter; UPI = uterine peristalsis imaging.

The subject underwent a 1-time, quick, anatomical (T1W sequence) 3T Siemens Prisma/Vida MRI scan (Fig. 1A and B) (15, 16, 17) to acquire the patient’s unique body-uterus geometry (Fig. 1C) while wearing eight patches containing 128 MRI-compatible fiducial markers around the abdomen and lower back.

### Electrical recording

After the MRI scan, customized BioSemi pin-type electrode patches (Fig. 1D) were applied to the same location on the body surface as the MRI fiducial marker patches, and an BioSemi analogue-to-digital converter box was used to record the body surface electrical signals (Fig. 1E). Then, the multichannel electromyography (EMG) signals were processed with a band-pass filter (0.01–0.05 Hz) (Fig. 1F). In each clinical visit, 2 EMG recording sessions were performed. In the first session, 20-min UPI scans were acquired without TVUS as a control. After that, 10-min UPI scans were acquired with simultaneous TVUS examination as a comparison.

### TVUS scan

A GE Voluson E6 US machine was used to evaluate the uterus including measurement of the endometrial lining and the ovaries including follicular measurements. The 10-minute TVUS scan was conducted concurrently with the UPI system, following the initial 20-minute UPI baseline study. Transvaginal US was performed by 1 of 2 trained sonographers while the patient was in dorsal lithotomy position. The sonographer initially obtained images of the adnexa and uterus before proceeding with the study scan. In the study scan, the probe was placed in contact with the cervix in the midline sagittal plane.

### Inverse computation

The UPI software was developed to solve the 3-dimensional Cauchy problem to formulate the uterine electrograms as electrical activities over time at each uterine site. Uterine surface EMGs specifically reflect the uterine surface electrical activities during UP. By identifying the time when the uterine EMGs reach the steepest negative slope, electrical activation sequence of the myometrium during a specific observation window was formed (Fig. 1G), where red regions represent areas experiencing the peristalsis and blue regions represent inactive areas of the uterus.

### Multiparametric quantifications of UP

The UPI postanalysis software was developed to quantify each peristalsis and generate the statistical UP report of each mapping session for each patient. Isochrone map (Fig. 1H) was formed on the basis of the activation sequence, where the gray colors denote the inactive uterine regions and the red as well as blue colors denote the early and late activation uterine regions, respectively, during peristalsis. Initiation (termination) sites were defined as the region experiencing the early (late) activation during UP. The common initiation (termination) sites were identified on the isochrone map, which included the cervical region, fundal region, left/right cornual region, and middle uterus.

The UP quantifications used included duration (second), magnitude (mV), frequency (per minute), and activation ratio (%) for each peristalsis. Uterine peristalsis duration was defined as the duration of a complete UP. As shown in Figure 1H, UP magnitude was defined as the mean peak amplitude of electrical potential over the uterine region experiencing activation during each UP. The UP frequency (per minute) was calculated as the number of peristalses over the imaging time (in minutes). As shown in Figure 1J, activation ratio was defined as the percentage of cumulative electrically activated area (decoded in red) over the entire uterine surface in each peristalsis. For example, 50% meant that the peristalsis wave activated 50% of the entire uterine surface.

### Statistical analysis

#### Analysis of demographic and clinical variables

Numeric variables among individuals who participated in the study were summarized using means and standard deviations. Categorical variables were summarized using frequencies and percentages.

#### Stationarity test

**Figure 2:**
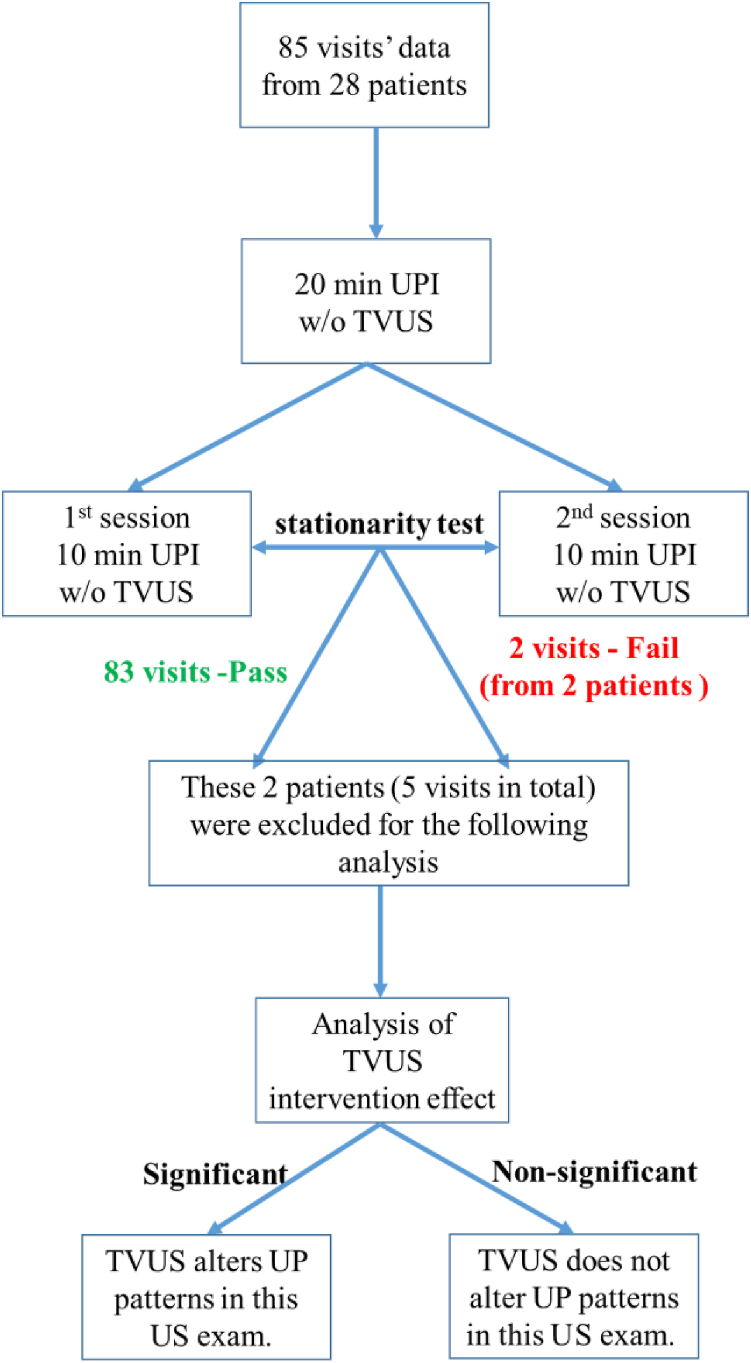
Statistical analysis flowchart to determine the stationarity test. TVUS = transvaginal ultrasound; UP = uterine peristalsis; UPI = uterine peristalsis imaging; US = ultrasound.

The primary outcomes of each UP were quantitative variables including duration (second), magnitude (mV), frequency (number per minute), and activation ratio (%). The first 20 minutes of electrical mapping session without TVUS was divided into 2 10-minute segments to test the stationarity of UP measurements. The Mann-Whitney *U* test was performed to determine whether there was a significant difference in each UPI parameter between two segments, and a *P* value of <.05 was considered statistically significant. If at least 1 UPI-indexed variable was identified as significantly different between the two periods, the UPI measurement would be considered as a nonstationary process (Fig. 2). This visit would be designated as a failed visit and excluded in analysis. If no significant difference was found between the two segments, the UP measurements were considered stable, and the passed visit was included in analysis.

#### Analysis of TVUS effect

The Wilcoxon matched-pairs signed rank test was performed to compare each averaged UPI-indexed variable of matched visits before and during US examinations.

## Results

Eighty-five visits were completed in 28 participants with regular menstrual cycles. Twenty-seven visits were not included because of incomplete data/technical issues or because participants dropped out or missed study visits. Table 1 demonstrates the demographic data and menstrual cycle history in all completed participants by phase of cycle. Missing studies were predominately in the periovulatory phase (11/28) because of its short duration and individual variability. At times, once the blood work from the visit was analyzed, the participant was found to be in the secretory phase, causing some patients to have more than one secretory visit without a periovulatory visit.

**Table 1:** Demographics of enrolled participants (N = 28) with regular menstrual cycles

Age, y	28.0 ± 4.6			
BMI, kg/m^2^	29.6 ± 8.1			
Race, n(%)	—			
White	14 (50.0%)			
Black	11 (39.2%)			
Asian	2 (7.1%)			
Other	1 (3.6%)			
Cycle length, d	28.0 ± 2.0			
Length of bleeding, n (%)	—			
3–5 d	17 (60.7%)			
6–7 d	7 (25.0%)			
Unknown	4 (14.3%)			
Phase	Menses (n = 21)	Proliferative (n = 21)	Ovulatory (n = 12)	Secretory (n = 31)
Estradiol (pg/mL)	37.3 ± 14.1	81.6 ± 29.8	180.0 ± 112.9	149.8 ± 93.5
Progesterone (ng/mL)	0.2 ± 0.1	0.2 ± 0.1	1.0 ± 1.1	7.3 ± 4.4
Endometrial thickness (mm)	3.6 ± 2.1	5.8 ± 2.3	9.8 ± 3.2	9.4 ± 2.8

*Note:* BMI = body mass index.

**Figure 3:**
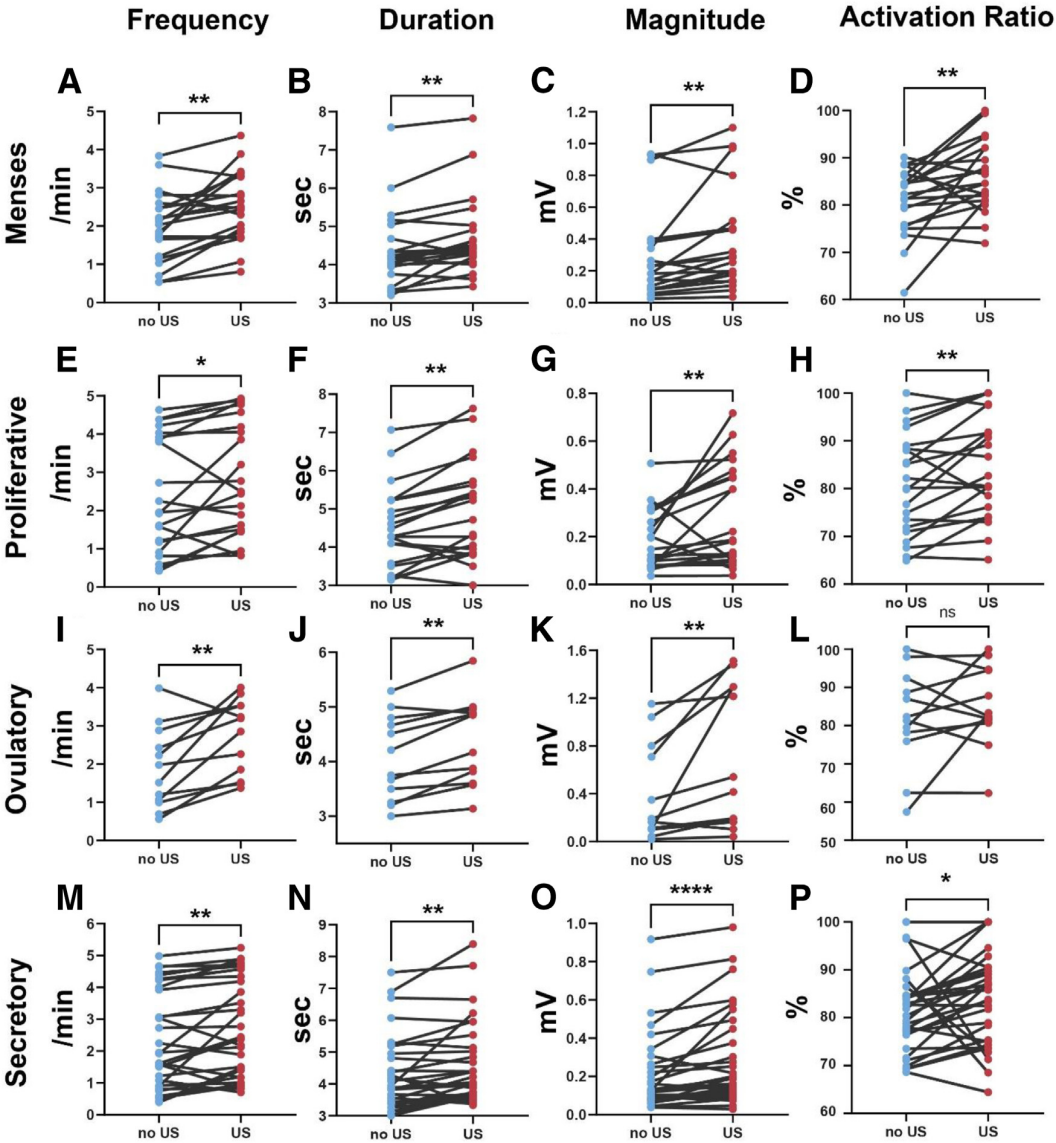
Pairwise comparison of transvaginal ultrasound (US) effect on the multiparametric uterine peristalsis quantifications before and during US examination. (**A–D**) Menses (n = 19). (**E–H**) Proliferative phase (n = 20). (**I–L**) Periovulatory phase (n = 11). (**M–P**) Secretory phase (n = 30). The pairwise t-paired test was performed to compare the mean value of the UP parameters for each visit in each patient. US = ultrasound. ∗*P*<.05, ∗∗*P*<.01, ∗∗∗*P*<.001, ∗∗∗∗*P*<.0001.

To assess whether there existed a statistically significant alteration in each of the UP parameters (duration, magnitude, frequency, and activation ratio) with the utilization of TVUS, we conducted a statistical comparison of UP patterns before and during the US examination across four phases in a single menstrual cycle. A total of 80 visits were included in analysis. Pairwise comparison analysis is demonstrated in Figure 3. In all phases, the frequency of peristalsis waves (measured in waves/min) was significantly higher after TVUS use. The duration of peristalsis waves (measured in seconds) was also significantly longer with TVUS use in all phases. In all phases, the magnitude of peristalsis waves (measured in mV) was significantly higher after TVUS use. Finally, the activation ratio (measured as a percentage) was significantly higher in all phases except for the periovulatory phase.

## Discussion

Our data demonstrate that UP waves are altered by the introduction of a TVUS probe, likely by mechanical pressure applied by the probe but also potentially from exposure to sound waves. The majority of the 80 visits had a change in at least one of the variables examined. Peristalsis waves had a significant increase in magnitude, frequency and duration during every phase of the menstrual cycle. Only the activation ratio during the periovulatory phase was not significantly different. Our results suggest a quantitative difference in peristalsis waves for participants undergoing TVUS.

Traditional 2-dimensional TVUS has been used for decades to image UP waves (3, 5, 10, 11), and newer technologies, including 4-dimensional imaging (18) as well as speckle tracking (19), have now been introduced to further characterize these waves. However, TVUS is only used for short time frames (typically 4–15 minutes) because of its invasive nature making it not capable of performing long-term comprehensive analysis (3, 19). Although US technology is considered easily reproducible and objective without obvious iatrogenic effects (20), only one study has evaluated the potential biologic effects from the equipment itself (21, 22, 23).

To our knowledge, this is the first study to evaluate whether TVUS may affect UP waves, which is important to determine whether it is an ideal imaging modality to quantify UP. A noninvasive system could better quantify these waves without causing iatrogenic changes. The UPI system our team has designed can create reconstructed uterine surface potentials to quantitatively image and measure 3-dimensional electrophysiologic activities of UP waves noninvasively without inducing changes.

The results presented here also demonstrate that TVUS appears to temporarily alter peristalsis waves differently at different phases. Hormonal fluctuations throughout the menstrual cycle contribute to inherent changes in peristalsis waves during each menstrual phase but may also explain why US affects the waves. Because each phase is hormonally distinct, US alters—by a mechanical effect and/or sound waves—peristalsis waves to a different degree throughout the menstrual cycle. For example, the steady increase in the levels of oxytocin and estrogen in the preovulatory follicles during the proliferative phase is thought to increase the frequency of peristalsis waves (24, 25). Because participants could have been early, mid, or late in the phase, this is likely the cause of the variability between participants in each of the outcomes. Similarly, during the periovulatory and secretory phases, the level of progesterone, a known muscle relaxant, increases as well as then decreases late in the secretory phase before menses and contributes to changes in peristalsis waves by having an antagonist effect on estrogen as well as oxytocin receptors (24, 25). Given that the participants had visits during different times in the periovulatory phase, the variability may cause a lack of group difference noted in the activation ratio before and during TVUS. Hormonal fluctuations because of the mechanical effect or through the sound waves of the TVUS probe may explain peristalsis wave alterations.

Previous studies have postulated that UP may affect embryo implantation and subsequent pregnancy loss (26, 27, 28). A study by Fanchin et al. (26) demonstrated that uterine contraction frequency, measured by TVUS, had an inverse relationship with implantation rates. Another study using TVUS demonstrated that specific peristalsis patterns predict more favorable in vitro fertilization outcomes in patients (27). More recently, Rees et al. (28) demonstrated uterine contractility differences in patients with and without adenomyosis using TVUS with speckle tracing. However, our study demonstrates that TVUS may have iatrogenic changes and, therefore, utilization of other imaging modalities may be better to study these important clinical implications. Future work will include using the UPI system, a noninvasive imaging modality, to study peristalsis waves in specific clinical situations such as before embryo transfer.

The main limitation of this study is the inability to determine when peristalsis waves return to physiologic patterns after TVUS use because data were only collected before and during TVUS use. Potential future work could include using the UPI system to evaluate peristalsis waves after the TVUS probe is removed or the use of wearable devices to monitor patterns over longer durations. Additionally, participants came on varying days of each phase; however, the menstrual cycle is known to be a dynamic process that changes each day, and thus, grouping by only the four phases may have caused increased variability. Furthermore, future studies using the transvaginal probe without turning it on would determine whether mechanical effects are distinct from sound wave effects. Finally, almost 6% of visits had to be excluded because of differences in the stationarity test, which may result in bias. The UPI system used in this study, with its noninvasive and quantitative capabilities, holds important potential for enhancing our understanding of the normal reproductive processes in the nonpregnant human uterus.

## Conclusion

In summary, data collected through the use of the UPI system have demonstrated that TVUS can, at least temporarily, alter UP waves in gynecologic participants with normal menstrual cycles. Changes include alterations in peristalsis frequency, duration, magnitude, and activation ratio. Because of the iatrogenic changes caused by TVUS, other noninvasive imaging modalities may be better suited for the study of UP.

## CRediT Authorship Contribution Statement

**Kelsey Anderson:** Writing – original draft, Project administration, Investigation, Formal analysis, Conceptualization. **Sicheng Wang:** Writing – review & editing, Writing – original draft, Visualization, Validation, Software, Resources, Project administration, Methodology, Investigation, Funding acquisition, Formal analysis, Data curation, Conceptualization. **Stephanie Pizzella:** Project administration, Data curation, Conceptualization. **Qing Wang:** Supervision, Project administration, Investigation, Funding acquisition, Conceptualization. **Yong Wang:** Writing – review & editing, Validation, Supervision, Resources, Project administration, Methodology, Investigation, Funding acquisition, Formal analysis, Conceptualization. **Valerie Ratts:** Writing – review & editing, Writing – original draft, Supervision, Resources, Project administration, Investigation, Funding acquisition, Conceptualization.

## Declaration of Interests

K.A. reports funding from March of Dimes Center Grant (22-FY14-486). S.W. has nothing to disclose. S.P. has nothing to disclose. Q.W. has nothing to disclose. Y.W. is a scientific consultant for Medtronic and EP Solution and reports grant R01HD104822 (PIs Wang/Schwartz/Cahill) and grants from the National Institutes of Health/National Institute of Child Health and Human Development (R01HD094381, PIs Wang/Cahill), Burroughs Wellcome Fund Preterm Birth Initiative (NGP10119, PI Wang), Bill & Melinda Gates Foundation (INV-037302, INV-005417, INV-035476, and 16INV-037302, PI Wang), and Institute of Clinical and Translational Science (5927, PI Wang) for the submitted work. V.R. Bill and Melinda Gates Foundation (INV-037302).

## Supplementary Data

Supplemental Figure 1 Flowchart of patient screening and enrollment.
