# Supplementary figures and images for: The use of transvaginal ultrasound alters physiologic uterine peristalsis in gynecologic participants
Source: F S Rep. 2024 Jun 25;5(3):296–303. doi: 10.1016/j.xfre.2024.06.004 (PMC11456659; doi:10.1016/j.xfre.2024.06.004)

Supplemental Figure 1


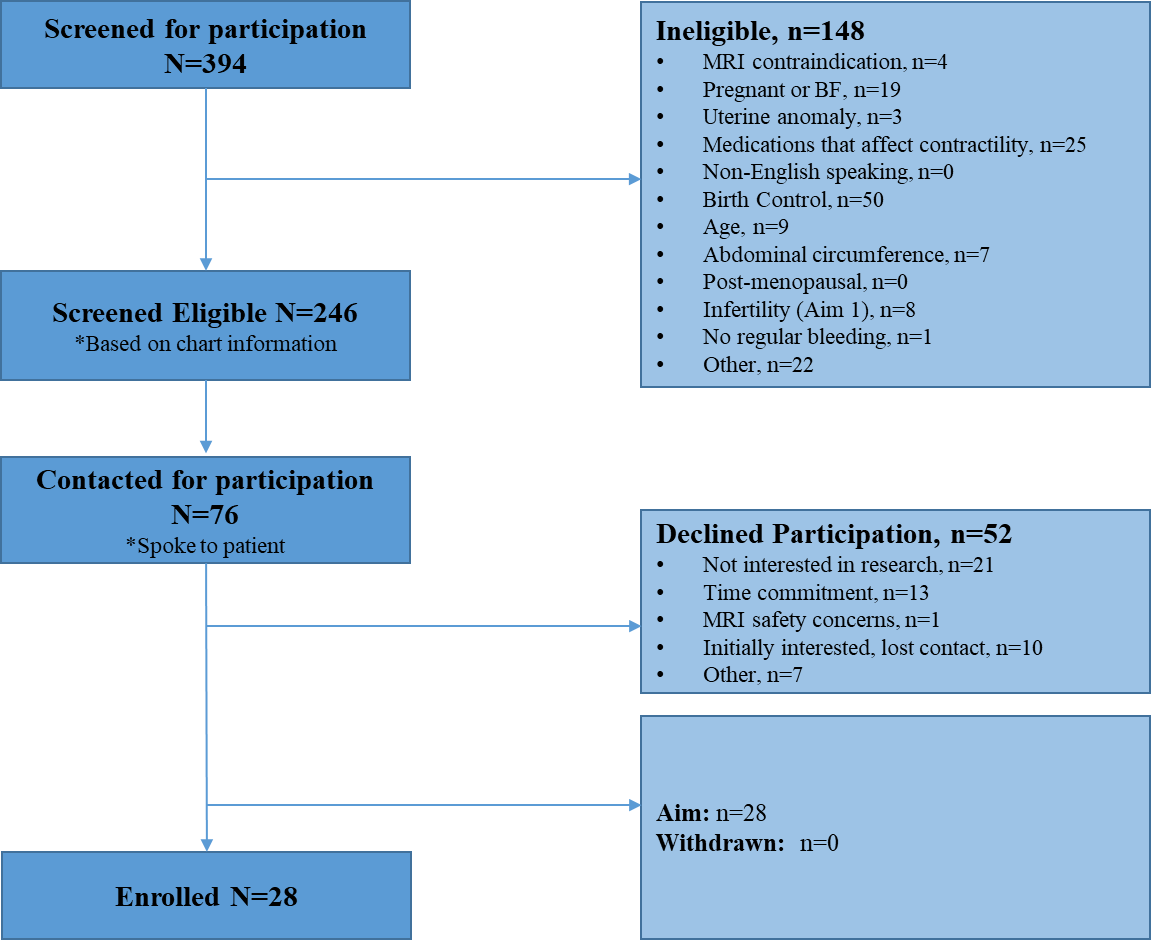

Supplement: Supplemental Figure 1 — Flowchart of patient screening and enrollment. [file mmc1.docx]
